# When academic leaders disconnect from social media in Finland: Situational rationality, silent resistance

**DOI:** 10.1177/09636625261469511

**Published:** 2026-08-03

**Authors:** Leena Ripatti-Torniainen, Karoliina Talvitie-Lamberg

**Affiliations:** 1 Faculty of Education and Culture, Tampere University, Finland; 2 Department of Language and Communication Studies, Faculty of Humanities and Social Sciences, University of Jyväskylä, Finland

**Keywords:** academic leadership, disconnection, science communication, situational analysis, social media, university communication

## Abstract

This qualitative study, conducted at Finland’s research universities, analyses high-profile academic leaders who have chosen to be disconnected from social media. We conducted individual interviews with 19 leaders and performed a situational analysis to investigate the rationale for abstaining from social media. Academic leaders rationalised disconnection by reasons related to personality, sociability, critical rationality, and health and security. The leaders’ intensive involvement in three social worlds intensified the individual rationale for disconnection and enabled the leaders to work successfully despite disconnection. These social worlds were research and science, sensitive academia, and insider networks in society. In the context of the managerial neoliberal university, its promotional communication culture, and competition for visibility, the leaders’ disconnection from social media has a dissident, resistant characteristic. The study contributes to prior research through its comprehensive approach to academic leaders’ operational environment. Future research should elaborate on the systemic approach to social media in universities.

## Introduction

Recent studies (Bucchi and Schäfer, 2025; Volk et al., 2024) indicate a need to analyse the relationship between social media and university management and leadership from systemic perspectives. University leaders have become more visible as academic figures and have come to exert a stronger influence on organisational communication than earlier (Fürst et al., 2023; Koivumäki and Wilkinson, 2022). Due to competition in neoliberal universities for commendatory visibility (Väliverronen et al., 2022) and the public demand for universities to interact with society (Besley, 2020), many universities in Finland have explicitly encouraged, even requested, managers and leaders to engage with social media via personal accounts. Contrary to these requests, there are academic leaders who make the choice to abstain from social media. Our study focuses on these high-profile academic leaders in Finland’s research-intensive universities who choose to disconnect from social media.

Formal academic leaders in Finland’s universities lead organisations that were transformed from state institutions to legally and financially independent entities via the Universities Act in 2009 (Finlex, 2009). The Humboldtian tradition of collegial decision-making was particularly strong in Finland through the tripartite representation of professors, other research and teaching staff, and students (Poutanen et al., 2022). In line with international development, the change in policy, legislation, and governance introduced strategic, top-down management; this led to the deterioration of collegial governance in Finland’s universities (Kohtamäki and Poutanen, 2025) and constitutes a delicate context for university communication and leaders’ social media use.

One of the features of social media is the blurring of the borders between private and professional lives (Fox and Bird, 2017; Mellado and Hermida, 2022; Ollier-Malaterre et al., 2013). For distinguished academic leaders in Finland’s universities, this blurring of lines extends from social media to professional loyalties and identities. Aligned with the legacy of collegial decision-making, academic leadership in Finland has historically rested on recognition of scientific peers. Academic leaders in Finland’s universities often identify themselves as researchers and members of the scientific community, which activates—discursively and interactively—a tense terrain in changed organisations. The strengthened managerial power in Finland’s universities (Silvén, 2023) faces sustained critique among the academic staff (Ministry of Education, 2012, 2016, 2021). In the hybrid media landscape, the occasionally escalated public uproar of the staff has specifically found expression on social media (Väliverronen et al., 2022).

In disconnection research, opting out of social media belongs under the umbrella term of digital disconnection that encompasses various temporary or permanent disengagement practices, such as withdrawing from digital platforms and deleting apps or social media accounts (Šimunjak, 2023). Former research has explained digital disconnection by a lack of skills and resources (Büchi and Hargittai, 2022; Hesselberth, 2018; Lüders and Brandtzæg, 2017). However, research is increasingly looking at disconnection as a conscious choice and, as some argue, as dissidence and resistance (Gangadharan, 2021; Portwood-Stacer, 2013; Vanden Abeele and Mohr, 2021). While the incentives to remain on social and digital platforms remain strong, disconnecting from social media platforms for the sake of individual digital well-being or as a counteraction to exploitative global data platforms has been on the rise (Enli and Fast, 2023). Preventing social media dependency is even seen as involved in responsible citizenship (Vanden Abeele and Mohr, 2021) as part of a neoliberal ideology of self-care (Docherty, 2021). Simultaneously, disconnection can indicate exceptional capacities or resources of the most privileged individuals (Fast et al., 2021) who are confident of their survival and success without digital visibility.

In highly educated populations such as academic leaders, disconnection from social media is unlikely to stem from a lack of opportunities. A prior study recognised digital disconnection early on among citizens with social, economic, and cultural capital (Oblak, 2013). As per previous digital disconnection research, academic leaders’ absence from social media could be assumed to be a voluntary, conscious act (Hesselberth, 2018; Karppi, 2018). Leaders’ disconnection from social media could manifest lifestyle politics (Portwood-Stacer, 2013) or search for authentic togetherness (Syvertsen and Enli, 2020). Disconnection from social media could include traits of micropolitical agency, dissidence, or resistance to the loss of privacy and the power of platforms (Johansson and Vinthagen, 2016; Talvitie-Lamberg et al., 2024; Vanden Abeele and Mohr, 2021), or even conscious use of power beyond datafied environments and their digital surveillance practices. These notions illuminate the weight of social, political, and public implications of disconnection from social media (Ross et al., 2024). As a choice made by distinguished academic leaders, disconnection from social media calls for a research-based understanding in today’s universities. In this qualitative study, we analyse the rationale and the broader communicative reality of academic leaders’ disconnection.

We ask the following research questions: (1) which rationale do academic leaders provide for their disconnection from social media? (2) Which features in universities characterise the broader situation of the leaders’ disconnection from social media?

## Data and data-collection methods

We conducted individual thematic interviews to collect data.^1^ Approval for the study was obtained after an ethical evaluation by the Human Sciences Ethics Committee at the University of Jyväskylä on 1 September 2021. Finland’s higher education sector consists of 13 universities, the National Defence University, and 22 universities of applied sciences. Of these, our focus is on the universities. These universities have the mission of independent academic research, research-based higher education, and the promotion of lifelong learning, interaction with society, and the social impact of university research findings and activities (Finlex, 2008, 2009). At a preliminary stage of the research, a research assistant scanned prospective social media accounts of rectors (presidents), vice rectors (vice presidents), chairpersons of university boards, chancellors, provosts, deans, and chairpersons of university collegia at all of Finland’s universities. Due to the significant distinction in size between Finland’s largest and smaller universities, vice deans at the largest university were also included. There is one vice dean in the data who has significant leadership responsibility. The names of the academic leaders were collected from the universities’ public internet sites.

The review by the research assistant found that from among the 195 leaders, 56 leaders did not have active accounts on Twitter (now X), Facebook, and Instagram. The first author sent personal invitations via email to these leaders to participate in the study. Of these, 21 leaders volunteered to participate, and interviews were conducted with 19 of them. The interviewees represented 11 universities and consisted of the following leadership categories: vice rectors (vice presidents), deans, chairs and vice chairs of university boards, chairs of university collegia, and vice deans (one vice dean in the data). The largest number of participants were deans, followed by vice rectors (vice presidents), chairs of university boards, chairs and vice chairs of university collegia, and the one vice dean in the data. In our study, universities with more than one participant were the largest universities in Finland. Almost all of our interviewees were full professors who had achieved leadership positions.

The first author conducted all interviews in Spring 2023 via Zoom (the video conferencing tool). Due to the leaders’ busy schedules, the duration of the interview was limited to 60 min. Only audio data were used for the study. The interviews were transcribed verbatim and pseudonymised before the analysis. The quotations included in this article were translated and slightly edited for readability by the first author.

We followed the ethical principles of research to respect the integrity of the research subjects (Finnish National Board on Research Integrity, 2019). Substantial information regarding the purpose of the study, a privacy notice, and an informed consent form was sent to the interviewees in advance via email. Consent was obtained orally and recorded at the beginning of each interview, and the documents were stored at the University of Jyväskylä’s protected research file storage. The interviewees introduced themselves in accordance with their preferences, thus guiding the level of professional and organisational details included in the data. Background information collected from the participants included their names and email addresses, which were stored in the protected research file storage and kept separate from the analysed material. The interviews revealed that abstaining from social media was a delicate issue in certain informants’ units. The reported results contain no references to the interviewee’s assumed age, assumed gender, title, discipline, job description, or any other personal, professional, or organisational details. For direct quotations, we refer to the informants by running numbers. These choices adhere to the guidelines of the General Data Protection Regulation (Council of Europe, 2016).

All interviews included the same main themes. The informants were asked to introduce themselves and speak about their motivation for participating in the study. Then followed the themes of social media disconnection; the use of digital platforms and media in general; key aspects of the interviewee’s work; communicative features of the interviewee’s work; and the interviewee’s thoughts, experiences, and dispositions regarding publicity, the public realm, public discourse, and public engagement. The interviews also provided time given for interviewees to comment on the questions or elaborate on their answers.

Furthermore, the interviews verged towards being active interviews (Holstein and Gubrium, 2003) in which the first author occasionally adopted the role of a conversation partner, for example, by occasionally expressing her instant interpretation of what the interviewee had said and requesting that the interviewee comment and elaborate on it. The active approach provided the interviewees with opportunities to ponder their social media disconnection with enhanced precision (Kvale and Brinkmann, 2009: 123–160). The interview design yielded coherent, internally consistent, and rich material.

The interviewed leaders are not unaware of social media. Even those of them who had never created social media accounts had knowledge and experiences of social media—for example, through family members’ or colleagues’ social media use. Most interviewees had created at least one social media account at some point—for example, in Facebook’s early days—and many passively maintained LinkedIn accounts. ‘I have a LinkedIn profile’, said Leader 3, ‘but except for accepting invitations I do nothing on LinkedIn. I have had a Twitter account since the start of Twitter. I posted twice. After that, I haven’t paid attention to Twitter’. Leader 9 reported,

I have one friend on Facebook. I had two friends, but I removed the other one. I think I am on Facebook because my [remaining] friend wants to use Messenger [an instant communication platform provided by Facebook]. I don’t follow Facebook at all.

‘I closed all accounts I had’, said Leader 2. ‘Well, I still have the LinkedIn account, but I don’t update it’. These extracts illustrate that most of the interviewees in our study had access to platforms but no desire to use social media.

## Analysis method and process

We used situational analysis (SA) (Clarke, 2007; Clarke et al., 2022) as the analysis method. As a social research method and an heir of social interactionism (Clarke, 2007), SA enabled us to pursue a comprehensive analysis of the situation in which academic leaders executed their choice of disconnection from social media. The SA enabled us to extend the analysis beyond individual choice to social dynamics and structural and material features of social media disconnection. The interview material was coded using Atlas.ti software in the following manner:

(a) Individual communicative factors: These include interview sequences of personal communicative dispositions, personal experiences with social media, and individual media consumption habits. For example, ‘I got used to it from the beginning that I didn’t consider myself to be such [a person] that I should be involved in social media’ (Leader 6).
(b) Social communicative factors: These include interview sequences of social interaction, communicative cultures, and socially esteemed and functional communication patterns. For example, ‘I somehow think that in the academic world, people who are very eager on social media are a little bit ridiculed. At least occasionally it is in the discussions whether they can be taken seriously anymore as researchers because they are on social media all the time’ (Leader 1).
(c) Structural communicative factors: These include interview sequences of established communicative procedures and conventions within and outside universities. For example, ‘We have arrangements. We have a communications and PR unit with several professionals and a vice dean with responsibility for societal interaction. Of course, I support them as much as I can, but there is this division of labour’ (Leader 10).
(d) Material communicative factors: These include interview sequences of material resources related to communication, such as assisting staff in service, and access to subscribed media content. For example, ‘I subscribe to four daily delivered newspapers’ (Leader 11) and ‘My secretary [. . .] filters topical issues for me. My secretary even brings them to my table, sends emails or calls me’ (Leader 1).

The SA enables a researcher to build an outline of the comprehensive situation, initially by producing ‘messy’ (Clarke, 2007: 86–88) notes and gradually constructing them into more organised ones (Clarke, 2007: 88–101). Table 1 displays an ordered document across the social, individual, structural, and material communicative factors in the interview data.

**Table 1.** Social, individual, structural, and material factors of academic leaders’ social media disconnection.

Individual communicative factors	—Face-to-face, bilateral interaction, and a discreet public profile are preferred —Focus on rational conversations —Heavy communicative workload (e.g. emails and meetings), careful, considerate approach to communicative tasks —Social media appears strange and repulsive —Valuing privacy —Social media causing health or security risks —Extensive consumption of news media
Social communicative factors	—Peer interaction in the scientific community as the communicative norm —Publicity expected to correspond with research expertise and research ethics —Sensitive communication environment in universities, causing a constant need to foresee prospective misunderstandings —Listening and conversation are emphasised as the communicative genres of leadership
Structural communicative factors	—Structured procedures regulating formal communication flows within universities —Established, often informal, communication relations within and outside universities, including politicians, authorities, and journalists —Division of labour in leadership teams in communication-related tasks —Extensive information and communication delivery via emails and meetings, yet only scarce notions of public discourse in universities —Lack of time for social media
Material communicative factors	—Abundant access to communication devices, software, and information flows, including political and governmental sources —Communication units and assisting administrative and research staff at service —Financial and lingual resources for wide consumption of subscription-based national and international news journalism

Table 1 serves as the foundation for our detailed analyses, which we will present next.

## Four threads explaining individual reasons for disconnection

To analyse academic leaders’ individual reasons for social media disconnection, we combined individual factors in the data into the four larger threads of (1) personality, (2) sociability, (3) rationality and criticality, and (4) health and security.

In *the personality thread*, the key reason for social media disconnection was a leader’s consideration of who they are. Leaders described their disconnection as arising from their personal characteristics: their self, identity, and temperament. ‘This is likely, I mean this is a personal characteristic [in me]’ (Leader 9). Disconnection from social media appeared to be a part of leaders’ considerate character and an intentionally discreet public profile. Leader 4 described long-running identity work and the resulting confidence in largely withdrawing from publicity:

I myself don’t feel that I would have a need to pursue publicity [. . .] Perhaps it’s part of the personality: who I am, what is my selfhood [. . .] I have had opportunities to go public a bit, but I found out that I perhaps prefer staying away. I don’t have a need to see my face out there immediately.

For certain leaders, disconnection from social media was one part of broader arrangements to draw a boundary between work and personal life. ‘My issues are my issues, and everyone doesn’t need to know everything. I have no need to perform a public profile in private life or at work’ (Leader 2). Careful consideration of public statements and messages to staff, as well as a preference for face-to-face, one-on-one encounters and conversations, was a repetitive feature in the interviews.

In *the sociability thread*, the emphasis moved from a leader’s self to a leader’s consideration of social interaction on social media platforms. Here, a key reason for disconnection was leaders’ experience or assumption of repulsive, unappealing interaction on social media. Leader 10, who had closed their social media accounts, listed three sociability-related reasons for the decision. First, Leader 10 was disappointed with ‘shouting’: social media users were leaving rational arguments at the mercy of opinions. The second reason was the ‘very disturbing’ communication culture on social media—for example, the requests to congratulate other users on their birthdays. The third reason was the exposure to people whose lives Leader 10 did not want to know about. Several other leaders referred to strong, uneasy reactions that social media sociability evoked in them. Although these aspects reflect a leader’s personality and could, therefore, fall into the personality thread above, the distinction from the personality thread is that here leaders did not emphasise their identities and selves but the manner in which other people socialised on social media. Several leaders expressed cultural criticism of sociability and communication on social media, as exemplified by Leader 9:

Why do people do that? [. . .] Why do people share indifferent events of their private lives? What is their motivation? Do they really want to make those issues public? All people have a life, and those same events and issues are experienced by everyone.

In *the rationality and criticality thread*, leaders saw disconnection from social media as a rational act and a reasonable outcome of critical consideration. Several leaders said they found ‘not enough reasons’ to engage with social media. ‘Our conduct should be knowledge-based in the university. I have read brain research and else enough to rationalise [my disconnection]. My own observations of my concentration and endurance support those results’ (Leader 1). Despite their formal leadership positions in university organisations, several leaders questioned the rationality of social media strategies and communication guidelines of their universities. ‘Our public image is not a measure of our impact on society’, said Leader 10, indicating that it was not reasonable or desirable to measure universities’ social media metrics. Over one-fourth of the leaders expressed critical notions regarding the societal implications of social media platforms on health, privacy, information security, or democracy. According to Leader 3, social media was ‘an unforeseen human experiment’ and there was not yet sufficient research on its implications.

Furthermore, lack of time, which was a prevalent topic in several interviews, appeared as a rational reason for social media disconnection. Even without social media presence, the leaders’ work involved a heavy digital and communicative workload. Meetings, emails, and scheduled tasks on digital platforms occupied vast parts of their days. Examples of weekly routines included working for 12–14 h on each working day, receiving approximately 500 emails each week, spending 2.5 h every weekday evening at home on compulsory email correspondence, extending working days to weekends, and conducting research in the late evenings and nights. Leader 11 said, ‘Should I additionally hang around on Twitter and Facebook and so on? There is no time for that. I work so much overtime in any way’.

*The health and security thread* refers to explicitly articulated health and security threat-related issues. Here, a key reason for disconnection was the assumed deteriorating effect that social media would have on mental or physiological health or security. ‘I prefer taking care of myself over burdening myself in one more way [i.e. social media]. There is enough burden already’, said one of the leaders.^2^ For another leader, the prospect of managing social media presence appeared as the straw that would break the camel’s back. ‘I can say it aloud: I can’t do that. I couldn’t do that. It is only barely that I survive’. Approaching an urgent tone, yet another leader described the impossibility of adding social media-related tasks in the completely scheduled days: ‘I never have time for coffee; never do I sit on a coffee break. I hardly get lunch. Recently, I have tried to secure [time for] lunch, but even today I didn’t catch it’.

The security issues referred to social media-mediated safety threats that had either occurred or would be likely to occur. One leader had been targeted every day with content disturbing enough to lose the next night’s sleep. According to the leader, they had thought, ‘What the hell am I doing here’, and withdrew from the social media channel. Another leader avoided social media due to harassment cases in the past.

Overall, the analysis of individual factors revealed substantial reasons for academic leaders’ social media disconnection. There is weight, particularly in the personality thread, which reveals social media disconnection being related to leaders’ self-concept, and in the health and security thread, which highlights the severe risks related to the use of social media by academic leaders. Each interviewed leader represented a unique combination of some or all of the four threads.

## The social, structural, and material factors of disconnection

Table 1 reveals that individual reasons were not the only factors responsible for the interviewed leaders’ social media disconnection. Social, structural, and material communicative factors in the leaders’ environment were also an integral part of the situation. The social factors presented in Table 1 reflect the strong influence of the scientific community on the interviewed leaders’ opinions regarding communication, and the characteristic communicative features of university organisations. The structural factors, again in Table 1, refer to procedures and established relations that exist to secure effective but not particularly public processes within university organisations and in universities’ external relations. Finally, the material factors in Table 1 reveal the leaders’ access to abundant material resources. Several leaders subscribed to global journalism outlets in several languages to remain updated and to expand on their judgement on topical issues. Despite their disconnection from social media, the interviewed leaders were connected to vast networks of national and international communication flows and had communication personnel, assisting staff, and experts in their service.

We build on these notions to identify the conditions that constitute the more permanent premises for social media disconnection in universities as compared to the factors in the interviewed leaders’ individual lives. Clarke (2007: 109–125) refers to this stage of SA as the analysis of social worlds, which implies analysing collectively maintained interpretations of the investigated phenomenon. Weaving social, structural, and material communicative factors (Table 1) together, we identified the interviewed leaders’ involvement in three social worlds that appear to make the disconnection from social media a rather logical choice. These worlds are (1) research and science, (2) sensitive academia, and (3) insider networks in society. Next, we separately analyse each world by highlighting the communicative conditions each world provides for social media disconnection.

## Research and science: Communicative conditions for disconnection

The interviewed leaders articulated strong identification with research, often suggesting that their management and leadership position were secondary to their professional identity as scientists and scholars. ‘Significant research results could be a reason to tweet’, said Leader 15, who did not have Twitter and LinkedIn accounts and who used Facebook only very narrowly for a hobby-related reason: ‘It hasn’t occurred to me to consider using Twitter in my [academic leader] role’.

Despite the often-expressed preference for discreet public profiles, the interviewed leaders often described their tight engagement with the characteristic publicness of the scientific community. Research expertise-based conversations and codevelopment with peers were ‘discussions that mattered’ (Leader 2).

Furthermore, the leaders often evaluated social media communication from a normative perspective of research ethics. The leaders emphasised that knowledge must be produced through ethically solid research evidence, with an awareness of the limits of what can be known and concluded. Discussion was expected to be rational and to respect the merits of the argument, and the time span of discussion was counted in years and even decades or longer. Several leaders regarded social media messages as too short to author a scientific argument. They considered social media as paralysing science and even deteriorating its authority. In the extract below, Leader 5 captured many of these notions:

I find it difficult to see that one could properly present scientific arguments in tweets [. . .] or on some Facebook or other platforms. I mean, I find it difficult to make a claim there and present support to the claim—empirical or theoretical or historical. [. . .] The natural medium of scientific argumentation is a medium that permits longer texts or argumentation. [. . .] Particularly suitable media are books.

A few leaders referred to the social discrediting in the scientific community of researchers, who were visible figures on social media. Because social media platforms provided publicity on grounds other than research merits, these researchers risked losing their credibility in academia. The interviewed leaders who referred to social discrediting themselves expressed esteem and encouragement for researchers who had found ways to use social media, thereby not discrediting anyone themselves.

In the social world of research and science, disconnection from social media appeared to be a logical, even natural, choice for many interviewees due to their strong commitment to the scientific community and its characteristic publicness among peers as well as to principles of scientific argumentation and research ethics. Disconnection from social media also enabled leaders to maintain their public image in the scientific community as researchers who did not compromise the principles of science for social media visibility despite their management and leadership tasks.

## Sensitive academia: Communicative conditions for disconnection

The interviews contained descriptions of sensitive internal communication environments in academic units. From the interviews, we distinguished two aspects relevant to social media disconnection: the emphasised need for sensitivity in the leadership of academic communities and the scarcity of references to public discourse.

Several leaders considered the communication environment in universities to be delicate, thereby intensifying their cautiousness regarding social media. ‘Even in a short text, such as an e-mail, I consider every word very carefully. I acknowledge that one must be very careful with social media’ (Leader 4). The awareness of a continuous possibility of misinterpretations and misunderstandings appeared to guide many leaders’ interactions with the research and teaching staff. Several leaders favoured face-to-face encounters and telephone calls and avoided writing unnecessary emails that ‘tend to lead issues into a wrong direction a little bit’, as Leader 14 said. A careful choice of words and a considerate public profile served to prevent tensions and to execute leadership in a fair and respectful manner. Leader 7 explained,

Many people retweet. [. . .] I find it dangerous. Someone [in the staff] might ask why I retweeted someone’s tweet and omitted retweeting another one. Might they think I prefer someone over someone else? Considering this, I feel I should follow everyone’s doings with great precision and be extremely careful of which tweets I redistribute. I kind of feel that to be active on social media would take enormous amounts of time because I should do social media so precisely and carefully.

In the interviews, we identified an unfamiliarity with public discourse that extended beyond cautiousness for social media. There appeared to be a boundary that was not willingly crossed: the boundary that marked the area of unknown responses and unknown turns of discussion that not only characterise social media but also general public discourse (Cayton, 2008). Apart from descriptions of staff gatherings and prepared public meetings, our material did not point towards a stabilised culture of public discourse in academic units. Formal meetings were carefully planned by the administration and backed by offstage negotiations, particularly before making important decisions that were prone to controversy. Leader 5 described,

One might say that issues are discussed in the preparatory stage, and time is set aside for conversations, especially if issues are difficult or complex, so that we have a shared understanding when those issues are officially decided. So . . . the non-public part is these conversations that enable us to make the decisions publicly, and everyone then understands what the decision is about.

Importantly, the interviews also highlighted differences between universities and units and suggested that certain leaders worked purposefully to develop internal communication.

It appears that interviewees’ notion of sensitive academic units made them consider disconnection from social media a rational choice. They believed that a leader’s active use of social media could intensify internal tensions and conflicts. Leader 4 said, ‘The bigger the audience, the more carefully the message must be thought out. [. . .] I am pleased to leave social media communications to those in the organisation who understand communication more than I do’. Leaders’ hesitation in public communication implies that professional communications units had a central role in their universities. It was through such units, through division of labour in the leadership teams, and through the personnel appointed in service of the leaders that university organisations appeared to execute communication effectively. This implies that there were structural and material arrangements that made disconnection affordable for leaders who chose disconnection. In addition, a few interviewees could ask for updates or even occasionally delegate social media-related tasks to research groups they formally led due to their permanent positions as full professors.

## Insider networks in society: Communicative conditions for disconnection

The interviewed leaders were tightly networked with governmental agencies, such as the Ministry of Education and Culture and the Research Council of Finland. These agencies are decisive in the funding of universities and science in Finland. According to the interviewees, customary communicative relationships also existed with parliament—both parliamentary committees and individual Members of Parliament. The relationships assumed two principal forms. First, the leaders were seasoned to act according to formal governmental procedures. Second, several of them personally knew prominent state officials and legislators. Occasionally, in our data, leaders appeared to be insiders who do business effectively behind the scenes, where decisions are ‘actually made’. Leader 8 explained,

I certainly know personally [. . .] all the people who are relevant to my work. I can call or send a message at any time about what is going on. [. . .] Even right now, I will next write a letter [the interviewee laughs] to my buddy [at the Ministry of Education and Culture] about what we think about this and that issue and that we hope it will proceed like this. And you know, they take us seriously. It has taken years to develop these relationships. But I don’t keep waiting to be informed; I’m digging for the information.

Close relations and access to the behind the scenes suggest that there was less need for using social media as an information source or for gaining visibility through social media presence. Almost every interviewee communicated confidence in directly accessing a newsroom when needed, at least with the help of in-house professional communications teams. However, in line with our interpretation of unestablished cultures of public discourse in academic units, there was not much information on engaging oneself in interaction with citizens through public discourse. Journalists emerged as key figures for such initiatives, as it is through their work that leaders aimed to influence public opinion. Leader 17 spoke about ‘a history of very informal channels’, referring to contacts that political decision-makers, senior officials, and prominent journalists had established with leading researchers and experts:

The relationships have been based on continuous contact, discussion, and involvement over many years. In recent years, or should I say in the last 20 years, I think that university departments or universities have more broadly tried to systematise channels for political decision-making and the media. I don’t know how well that worked out, though. I would argue that the key contacts are still based on informal bilateral contacts, either between political decision-makers, senior officials, and individual researchers, or between the leading media outlets and individual researchers.

Involvement in insider networks appeared to provide an important condition for academic leaders’ social media disconnection. Access to information, conversations, and influence in society was secured in insider networks through personal relationships and participation in processes and procedures of the parliament and governmental agencies.

## Discussion of results and conclusion

The results of this study reveal that academic leaders’ disconnection from social media was based on a bundle of individual reasons and on leaders’ involvement in social worlds, which intensified their individual rationale for disconnection and enabled them to work successfully despite social media disconnection. Previous research on knowledge-intensive work has identified variation regarding how individuals experience the impact of digital media in their lives (Karlsen and Ytre-Arne, 2022). The leaders interviewed in our study identified or assumed a heavy, burdensome impact of social media on their individual lives. Our results also reveal the interviewees’ privileged position of working in an environment that adapted communication to their preferences. Indicating digital privilege, the leaders appeared to be confident regarding their survival and success without digital visibility (Fast et al., 2021). Each identified social world in the data—research and science, sensitive academia, and insider networks in society—provided communicative conditions that made social media disconnection an affordable, even a reasonable, choice. With these results, our study contributes to digital disconnection research by bringing forward the specificities of social media disconnection in research-intensive universities, particularly regarding well-resourced individuals who have well-secured positions in universities.

We consider the social worlds in our data as signalling an underlying, socially maintained institution (Helmke and Levitsky, 2004; Schmidt, 2010) in Finland’s universities. Conceptually, an institution refers to norms, procedures, and discourses that are maintained in social interaction. Due to its socially and interactionally maintained character, a socially esteemed institution, such as the institution of science in universities, can enable participants to live up to another trajectory of their work and organisation than the one promoted in organisational strategy (Helmke and Levitsky, 2004: 727; Mahoney and Thelen, 2010). A discourse that draws on a socially championed institution can prevent a divergent organisational effort from taking root (Buchan and Simpson, 2024; McCammon, 2022). The social institution suggested by our results guards the priority of research in universities and the autonomy of research-based thinking; favours conversations between peers and specialists; and operates in society through established procedures and bilateral relations with governmental authorities, decision-makers, and journalists. For those who are socialised into these communication modes, engagement with social media and the general public may not appeal as a particularly socially functional and rewarding means of communication.

Previous studies on social media in science communication research have identified issues that prevented social media use in universities, such as the anticipation of peers’ negative responses (Entradas and Bauer, 2019), the risk of losing one’s credibility (Rödder, 2012), the hesitation of having to deal with uncontrollable publicity, the ethical issues of social media (Metag, 2021), and harassment and security threats to researchers (Väliverronen and Saikkonen, 2021), which have also been identified in our study. Our study, with its emphasis on formal academic leaders and the comprehensive communicative reality of their work, is a step towards a systemic understanding of relations between the previously identified factors and the broad operational environment of universities today.

Previous digital disconnection research has highlighted the growing trend of micropolitical resistance to platforms and their attention economies and emphasised social media abstention as dissidence (Kaun and Treré, 2020) and everyday resistance (Gangadharan, 2021; Johansson and Vinthagen, 2016; Talvitie-Lamberg et al., 2024). In the context of the managerial, neoliberal university (Kohtamäki and Poutanen, 2025), its promotional communication culture (Väliverronen et al., 2022), and competition for commendatory visibility (Bucchi and Schäfer, 2025), the interviewed academic leaders indeed appear as somewhat dissident, resistant figures. This is the case, for example, when they rationalise their abstaining from social media by defending the authority of science that social media threatens to deteriorate or when they deliberately practise collegiality as leaders of universities governed by a centralised management. Instead of voicing their critical thinking via social media (see Volk et al., 2024), the criticism of our interviewed leaders became evident in their absence from social media. Their critical voice, which their silence on social media platforms revealed to us, is worth recognition in science communication research.

Socially maintained institutions are not exclusive; instead, legacy institutions and emergent institutions often exist discursively and interactionally side by side in organisations, particularly during transitional periods (Buchan and Simpson, 2024). During the time of our interviews, there were other leaders in the same universities who posted extensively on social media. A limitation of our study is that we do not acknowledge incentives for social media use in the same organisations in which our interviewees interpreted implicit support for social media disconnection. Another question that remains beyond the scope of our study is the influence of academic leaders who choose disconnection on universities’ strategic communication. Concurrently with the interviewed leaders’ years of absence from social media, university staff have used social media to criticise the management and leadership of Finland’s universities (e.g. Väliverronen et al., 2022). To what extent these distinct strategies of agency—engagement with and absence from social media—address each other is worth investigating. Universities should be aware of the socially and politically relevant processes that are stratified throughout social media, such as the datafied construction of knowledge and epistemic authority (Bartsch et al., 2025), social processes and groups (Møller Hartley et al., 2023), and public agency (Ojala and Ripatti-Torniainen, 2024; Starr, 2021). To obtain a systemic understanding of research universities and social media, further studies should bring these parallel settings together and extend the analysis to related power struggles.

## Supplemental Material

sj-docx-1-pus-10.1177_09636625261469511 – Supplemental material for When academic leaders disconnect from social media in Finland: Situational rationality, silent resistance Supplemental material, sj-docx-1-pus-10.1177_09636625261469511 for When academic leaders disconnect from social media in Finland: Situational rationality, silent resistance by Leena Ripatti-Torniainen and Karoliina Talvitie-Lamberg in Public Understanding of Science

## Data Availability

The transcribed interviews are stored in the secure research data storage system of the University of Jyväskylä. Due to ethical requirements related to participants’ consent, the interview data are not available to external users.

## Notes

1. The interviews with academic leaders constitute one subdata of a broader research project investigating disconnection from social media. The other three subdata groups are distinguished journalists, young adults (mostly with higher education degrees or ongoing studies at university), and immigrants with a refugee background. The analysis presented in this article is the first published study of the project.

2. We have excluded the coded names of all leaders in this text section due to the sensitivity of the interview speech.

## References

[bibr1-09636625261469511] Bartsch A Neuberger C Stark B , et al. (2025) Epistemic authority in the digital public sphere: An integrative conceptual framework and research agenda. Communication Theory 35(1): 37–50.

[bibr2-09636625261469511] Besley JC (2020) Five thoughts about improving science communication as an organizational activity. Journal of Communication Management 24(3): 155–161. 10.1108/JCOM-03-2020-0022

[bibr3-09636625261469511] Bucchi M Schäfer MS (2025) Tensions in the public communication by scientists and scientific institutions: Sources, dimensions, and ways forward. Public Understanding of Science. Epub ahead of print 29 May. 10.1177/09636625251343507 40439249

[bibr4-09636625261469511] Buchan L Simpson B (2024) Collaborative leadership: A processual approach. In: Knights D Liu H Smolović-Jones O , et al. (eds) The Routledge Critical Companion to Leadership Studies. Routledge, pp.80–90. Available at: https://learning.oreilly.com/library/view/the-routledge-critical/9781040024973/xhtml/ch07.xhtml#chapter7

[bibr5-09636625261469511] Büchi M Hargittai E (2022) A need for considering digital inequality when studying social media use and well-being. Social Media & Society 8(1): 1–7. 10.1177/20563051211069125

[bibr6-09636625261469511] Cayton MK (2008) What is public culture? Agency and contested meaning in American culture—an introduction. In: Shaffer MS (ed.) Public Culture: Diversity, Democracy, and Community in the United States. University of Pennsylvania Press, pp.1–25.

[bibr7-09636625261469511] Clarke A (2007) Situational Analysis: Grounded Theory after the Postmodern Turn. Sage.

[bibr8-09636625261469511] Clarke A Friese C Washburn RS (2022) Situational Analysis in Practice: Mapping Relationalities across Disciplines. Routledge.

[bibr9-09636625261469511] Council of Europe (2016) Council Regulation (EC) 2016/679 on general data protection (GDPR) (2016). Regulation of the European Parliament and of the Council of 27 April 2016 on the protection of natural persons with regard to the processing of personal data and on the free movement of such data, and repealing directive 95/46/EC. Official Journal L(119): 1–156. Available at: https://eurlex.europa.eu/eli/reg/2016/679/oj

[bibr10-09636625261469511] Docherty N (2021) Digital self-control and the neoliberalization of social media well-being. International Journal of Communication 15: 3827–3846.

[bibr11-09636625261469511] Enli G Fast K (2023) Political solutions or user responsibilization? How politicians understand problems connected to digital overload. Convergence 29(3): 675–689.

[bibr12-09636625261469511] Entradas M Bauer MW (2019) Bustling public communication by astronomers around the world driven by personal and contextual factors. Nature Astronomy 3(2): 183–187.

[bibr13-09636625261469511] Fast K Lindell J Jansson A (2021) Disconnection as distinction: A Bourdieusian study of where people withdraw from digital media. In: Jansson A Adams PC (eds) Disentangling: The Geographies of Digital Disconnection. Oxford University Press, pp.61–90.

[bibr14-09636625261469511] Finlex (2008) Laki maanpuolustuskorkeakoulusta [National Defence University Act]. Available at: https://www.finlex.fi/fi/lainsaadanto/2008/1121 (accessed 30 December 2025).

[bibr15-09636625261469511] Finlex (2009) Universities act. Available at: https://www.finlex.fi/en/legislation/translations/2009/eng/558 (accessed 30 December 2025).

[bibr16-09636625261469511] Finnish National Board on Research Integrity (2019) The Ethical Principles of Research with Human Participants and Ethical Review in the Human Sciences in Finland. Finnish National Board for Research Integrity TENK. Available at: https://tenk.fi/sites/default/files/2021-01/Ethical_review_in_human_sciences_2020.pdf (accessed 17 March 2025).

[bibr17-09636625261469511] Fox A Bird T (2017) The challenge to professionals of using social media: Teachers in England negotiating personal-professional identities. Education and Information Technologies 22(2): 647–675.

[bibr18-09636625261469511] Fürst S Schäfer MS Vogler D , et al. (2023) Beyond the news media logic? Analyzing the social media orientation of university leadership. Schweizerische Zeitschrift Für Soziologie 49(3): 567–588. 10.2478/sjs-2023-0027

[bibr19-09636625261469511] Gangadharan S (2021) Digital exclusion: A politics of refusal. In: Bernholz L Landemore H Reich R (eds) Digital Technology and Democratic Theory. University of Chicago Press, pp.113–140.

[bibr20-09636625261469511] Helmke G Levitsky S (2004) Informal institutions and comparative politics: A research agenda. Perspectives on Politics 2(4): 725–740. 10.1017/S1537592704040472

[bibr21-09636625261469511] Hesselberth P (2018) Discourses on disconnectivity and the right to disconnect. New Media & Society 20(5): 1994–2010. 10.1177/1461444817711449 PMC6256718 30581360

[bibr22-09636625261469511] Holstein JA Gubrium JF (2003) Active interviewing. In: Holstein JA Gubrium JF (eds) Postmodern Interviewing. Sage, pp.66–80.

[bibr23-09636625261469511] Johansson A Vinthagen S (2016) Dimensions of everyday resistance: An analytical framework. Critical Sociology 42(3): 417–435.

[bibr24-09636625261469511] Karlsen F Ytre-Arne B (2022) Intrusive media and knowledge work: How knowledge workers negotiate digital media norms in the pursuit of focused work. Information, Communication & Society 25(15): 2174–2189. 10.1080/1369118X.2021.1933561

[bibr25-09636625261469511] Karppi T (2018) Disconnect: Facebook’s Affective Bonds. University of Minnesota Press.

[bibr26-09636625261469511] Kaun A Treré E (2020) Repression, resistance, and lifestyle: Charting (dis)connection and activism in times of accelerated capitalism. Social Movement Studies 19(5–6): 697–715.

[bibr27-09636625261469511] Kohtamäki V Poutanen M (2025) External university autonomy evaluations: The new autonomy ecology of Finnish universities. Journal of Higher Education Policy and Management 47(6): 649–668. 10.1080/1360080X.2025.2470697

[bibr28-09636625261469511] Koivumäki K Wilkinson C (2022) ‘One might tweet just for money’: Organisational and institutional incentives for researchers’ social media communication and public engagement practices. Studies in Communication Sciences 22(3): 471–491. 10.24434/j.scoms.2022.03.3205

[bibr29-09636625261469511] Kvale S Brinkmann S (2009) Interviews: Learning the Craft of Qualitative Research Interviewing. 2nd ed. Sage.

[bibr30-09636625261469511] Lüders M Brandtzæg PB (2017) “My children tell me it’s so simple”: A mixed-methods approach to understand older non-users’ perceptions of social networking sites. New Media & Society 19(2): 181–198. 10.1177/1461444816667862

[bibr31-09636625261469511] Mahoney J Thelen K (2010) Explaining Institutional Change: Ambiguity, Agency, and Power. Cambridge University Press.

[bibr32-09636625261469511] McCammon H (2022) Discursive opportunity structure. In: Snow D Della Porta D McAdam D , et al. (eds) The Wiley-Blackwell Encyclopedia of Social and Political Movements. Wiley, pp.1–3. 10.1002/9780470674871.wbespm073.pub2

[bibr33-09636625261469511] Mellado C Hermida A (2022) A conceptual framework for journalistic identity on social media: How the personal and professional contribute to power and profit. Digital Journalism 10(2): 284–299.

[bibr34-09636625261469511] Metag J (2021) Tension between visibility and invisibility: Science communication in new information environments. Studies in Communication Sciences 21(1): 129–144. 10.24434/j.scoms.2021.01.009

[bibr35-09636625261469511] Ministry of Education and Culture (2012) Yliopistolakiuudistuksen vaikutusten arviointi [Assessment of the impacts of the Universities Act Reform]. Opetus- ja kulttuuriministeriön julkaisuja [Publications of the Ministry of Education and Culture]. Available at: http://urn.fi/URN:ISBN:978-952-263-140-4

[bibr36-09636625261469511] Ministry of Education and Culture (2016) Yliopistolakiuudistuksen vaikutusten arviointi [Assessment of the Impacts of the Universities Act Reform]. Opetus- ja kulttuuriministeriön julkaisuja [Publications of the Ministry of Education and Culture]. Available at: http://urn.fi/URN:ISBN:978-952-263-420-7

[bibr37-09636625261469511] Ministry of Education and Culture (2021) Yliopistoautonomia [University autonomy]. Opetus- ja kulttuuriministeriön julkaisuja [Publications of the Ministry of Education and Culture]. Available at: http://urn.fi/URN:ISBN:978-952-263-885-4

[bibr38-09636625261469511] Møller Hartley J Bengtsson M Schjøtt HA , et al. (2023) Researching publics in datafied societies: Insights from four approaches to the concept of “publics” and a (hybrid) research agenda. New Media & Society 25(7): 1668–1686. 10.1177/14614448221194752

[bibr39-09636625261469511] Oblak ČT (2013) Slovenians offline. Czech Sociological Review 49(6): 927–950.

[bibr40-09636625261469511] Ojala M Ripatti-Torniainen L (2024) Where is the public of ‘networked publics’? A critical analysis of the theoretical limitations of online publics research. European Journal of Communication 39(2): 145–160. 10.1177/02673231231210207

[bibr41-09636625261469511] Ollier-Malaterre A Rothbard NP Berg JM (2013) When worlds collide in cyberspace: How boundary work in online social networks impacts professional relationships. The Academy of Management Review 38(4): 645–669. 10.5465/amr.2011.0235

[bibr42-09636625261469511] Portwood-Stacer L (2013) Media refusal and conspicuous non-consumption. New Media & Society 15(7): 1041–1057. 10.1177/1461444812465139

[bibr43-09636625261469511] Poutanen M Tomperi T Kuusela H , et al. (2022) From democracy to managerialism: Foundation universities as the embodiment of Finnish university policies. Journal of Education Policy 37(3): 419–442. 10.1080/02680939.2020.1846080

[bibr44-09636625261469511] Rödder S (2012) The ambivalence of visible scientists. In: Rödder S Franzen M Weingart P (eds) The Sciences’ Media Connection—Public Communication and Its Repercussions (Sociology of the Sciences Yearbook). Vol. 28. Springer, pp.155–177. Available at: https://link.springer.com/book/10.1007/978-94-007-2085-5

[bibr45-09636625261469511] Ross MQ Gilbert A Klingelhoefer J , et al. (2024) Mapping a pluralistic continuum of approaches to digital disconnection. Media, Culture & Society 46(4): 851–862. 10.1177/01634437241228

[bibr46-09636625261469511] Schmidt VA (2010) Taking ideas and discourse seriously: Explaining change through discursive institutionalism as the fourth ‘new institutionalism’. European Political Science Review 2(1): 1–25. 10.1017/S175577390999021X

[bibr47-09636625261469511] Silvén P (2023) Managerialistinen siirtymä: Managerialismi julkisoikeudellisten monialayliopistojen johtosäännöissä [The shift toward managerialism: Managerialism in the governance regulations of public multidisciplinary universities]. Tiedepolitiikka 48(4): 18–32. 10.58957/tp.135924

[bibr48-09636625261469511] Šimunjak M (2023) You have to do that for your own sanity: Digital disconnection as journalists’ coping and preventive strategy in managing work and well-being. Digital Journalism 13(2): 328–347. 10.1080/21670811.2022.2153711

[bibr49-09636625261469511] Starr P (2021) The relational public. Sociological Theory 39(2): 57–80. 10.1177/07352751211013244

[bibr50-09636625261469511] Syvertsen T Enli G (2020) Digital detox: Media resistance and the promise of authenticity. Convergence 26(5–6): 1269–1283. 10.1177/1354856520934696

[bibr51-09636625261469511] Talvitie-Lamberg K Lehtinen V Valtonen S (2024) Tactics of invisibility: How people in vulnerable positions make datafied everyday life livable. New Media & Society 26(9): 5445–5465. 10.1177/14614448221136077

[bibr52-09636625261469511] Väliverronen E Saikkonen S (2021) Freedom of expression challenged: Scientists’ perspectives on hidden forms of suppression and self-censorship. Science, Technology & Human Values 46(6): 1172–1200. 10.1177/0162243920978303

[bibr53-09636625261469511] Väliverronen E Sihvonen T Laaksonen S-M , et al. (2022) Branding the “wow-academy”: The risks of promotional culture and quasi-corporate communication in higher education. Studies in Communication Sciences 22(3): 493–513. 10.24434/j.scoms.2022.03.3285

[bibr54-09636625261469511] Vanden Abeele MM Mohr V (2021) Media addictions as Apparatgeist: What discourse on TV and smartphone addiction reveals about society. Convergence 27(6): 1536–1557. 10.1177/13548565211038539

[bibr55-09636625261469511] Volk SC Vogler D Fürst S , et al. (2024) The plurivocal university: Typologizing the diverse voices of a research university on social media. Public Understanding of Science 34(3): 270–290. 10.1177/09636625241268700 PMC11927015 39177096

