# Supplementary material for: When academic leaders disconnect from social media in Finland: Situational rationality, silent resistance
Source: Public Underst Sci. 2026 Aug 3;35(8):918–33. doi: 10.1177/09636625261469511 (PMC13696776; doi:10.1177/09636625261469511)
Supplement: Supplementary file 1 [file sj-docx-1-pus-10.1177_09636625261469511.docx]

**Supplemental material: Interview Guide**

**Article title:**

Academic leaders’ disconnection from social media in Finland: Silent resistance and situational rationality

**Authors:**

1) Leena Ripatti-Torniainen
   Faculty of Education and Culture 
   Tampere University, Finland
   ORCID: orcid.org/0000-0003-1742-411X
2) Karoliina Talvitie-Lamberg
   Faculty of Humanities and Social Sciences
   University of Jyväskylä, Finland
   ORCID: orcid.org/0000-0003-0697-0385


   Corresponding author: Leena Ripatti-Torniainen
   Email: leena.ripatti-torniainen@tuni.fi

**Content:**

- Interview Guide, original in Finnish
- Interview Guide, English translation

**Interview Guide, Original in Finnish**

**Mediakieltäytyjät-hankkeen haastattelurunko (yliopistojohtajat, huhtikuu 2023)**

1. **Esittäytyminen**

   -Millä tavalla haluaisit esitellä itsesi tämän tutkimuksen osallistujana? Ei nimeä.

   -Kutsuimme sinut somen ei-käyttäjiä kokoavaan tutkimukseen. Mikä aiheessa tuntui tulevan niin lähelle, että päätit osallistua?
2. **Somen ulkopuolelle jättäytyminen: keskeisiä syitä ja laajempia taustoja haastateltavan itsensä kertomana**

   **-**Milloin tulit ajatelleeksi ja huomanneeksi, että olet somen ulkopuolella? Mitkä tekijät olivat johtaneet tilanteeseen?

   -Miten kuvailisit somea - esimerkiksi mitä some-kanavia tunnistat nimeltä

   -Millaisista syistä somen ulkopuolella pysyminen on vakiintunut kohdallasi? Miksi on sinulle merkityksellistä tai tärkeää, että jatkat somen ulkopuolella olemista?
3. **Some ja digitaaliset alustat yliopistojohtajan työssä**

   -Millä tavalla käytät digitaalisia alustoja, tietoverkkoja ja mahdollisesti somea työssäsi?

   -Miten pyrit hallitsemaan digitaalista läsnäoloasi?

   -Millaisen aseman some ja digitaaliset alustat näyttävät saaneen yliopistojohtajien työssä?
4. **(Työ)elämäsi keskeisiä piirteitä itsesi kertomana**

   -Tavanomaisen työviikon kuvaus: Mitä tapahtuu, missä ja keiden kanssa?

   -Edellisiä täydentämään: tavanomaisen kuukauden ja tavanomaisen vuoden näkökulma

   -Yleisesti elämän keskeiset kiintopisteet: Mitkä asiat ja tekijät ohjaavat elämääsi ja toimintaasi?
5. **Somen ulkopuolella: ulkopuolisuus ja osallisuus**

   -Millaisia ulkopuolelle jäämisen/ ulkopuolisuuden kokemuksia ja tilanteita some-ulkopuolisuus on aiheuttanut työssäsi?

   **-**Konkreettisia tilanteita, joissa syntynyt ulkopuolisuuden tunteita – ulkopuolisuutta mihin ja keneen?

   -Konkreettisia tilanteita, joissa somen ei-käyttö on vaikeuttanut elämää - miten ja millaisin seurauksin?

   -Miten olet käytännössä toiminut näissä tilanteissa?

   -Konkreettisia tilanteita, joissa olet ollut tyytyväinen some-ulkopuolisuuteesi?

   -Oletko joskus harkinnut alkavasi käyttää somea – millaisissa tilanteissa ja asiayhteyksissä ajatus on syntynyt? Mikä on saanut sinut pysymään valinnassasi olla käyttämättä somea?
6. **Viestintä yliopistojohtajan työssä**

   -Viestintä yliopiston sisällä ja yliopiston ulkopuolelle on keskeinen osa akateemisten johtajien työtä. Millä tavalla poissaolosi somesta heijastuu arviosi mukaan viestintätoiminnassasi?

   -Miten kuvailisit osallisuuttasi, toimijuuttasi ja verkostoitasi?

-Millaisia keinoja sinulla on saada muiden tietoisuuteen tärkeinä pitämiäsi asioita ja näkökulmia ja mahdollisesti keskustella niistä muiden kanssa?

7. **Julkinen toiminta yliopistojohtajan työssä**

   -Missä hahmotat sellaista julkisuutta, jossa käsitellään sinulle tärkeitä asioita? Millä tavalla voit vaikuttaa tuohon julkisuuteen - entä olla tuottamassa tärkeille asioille näkyvyyttä ja vaikuttavuutta itse?

   -Miten kuvailisit yksityisiä ja ammatillisia mahdollisuuksiasi toimia tärkeinä pitämiesi asioiden eteen yhteiskunnassa? Miten somen ulkopuolella oleminen heijastuu toimintatilaasi oman arviosi mukaan?

-Miten määrittelet julkisuuden nyky-yhteiskunnassa?

-Millaisiin itsellesi tärkeisiin yhteisöihin tunnet kuuluvasi?

-Millä tavoilla voit vaikuttaa oman elämäsi kannalta merkityksellisiin asioihin?

-Mistä päiväsi puheenaiheet tulevat?

8. **Ajatukset tulevaisuutta kohti**

   -Some-suhde sekä oman osallisuuden ja toimintamahdollisuuksien näkymiä

   -Mitä haastateltava haluaisi vielä itse nostaa esiin haastattelun teemasta

   -Kiitokset haastatteluun osallistumisesta

**Interview guidelines, an English translation**

**Interview Guide for the Media Refusers Research Project (Academic Leaders, April 2023)**

1. **Introduction**

- How would you like to introduce yourself as a participant in this study? Please do not mention your name.
- You were invited to participate in a study focusing on people who do not use social media. What aspects of this topic resonated with you personally and motivated you to take part?

2. **Opting Out of Social Media: Key Reasons and Contexts**

- When did you first become aware that you were outside social media? What factors do you think contributed to this?
- How would you describe social media? For example, which platforms can you name.
- What reasons have led you to continue staying outside social media? Why is it important or meaningful for you to maintain this position?

3. **Social media and digital platforms in academic leadership**

- In what ways do you use digital platforms, online networks, and possibly social media in your work?
- How do you seek to manage your digital presence?
- What kind of role do social media and digital platforms appear to have assumed in the work of university leaders?

4. **Key Aspects of (Professional) Life as an Academic Leader**

- Please describe your typical work week: What do you do, where, and with whom?
- Please elaborate on this description by also reflecting on a typical month and year.
- What kinds of influences or circumstances do you feel shape your life choices and everyday actions?

5. **Being Outside Social Media: Experiences of Inclusion and Exclusion**

- In what ways has your non-participation in social media contributed to experiences of exclusion in your professional context?
- Can you describe specific situations in which you have experienced exclusion—exclusion from what, and in relation to whom?
- Can you describe specific situations in which not using social media has made your work or everyday life more difficult? In what ways, and with what consequences?
- How have you responded or acted in these situations?
- Can you describe specific situations in which you have felt satisfied or content with being outside social media?
- Have you ever considered starting to use social media? In what situations or contexts has this thought arisen? What has led you to maintain your decision not to use social media?

6. **Communication in academic leadership**

- Communication within the university and with external audiences and stakeholders is a central part of academic leadership. In your view, how is your absence from social media reflected in your communication practices?
- How would you describe your participation in societal and professional contexts, your sense of agency, and the networks you are part of?
- What strategies or means do you use to communicate issues and perspectives you consider important, and to engage others in dialogue within your professional context?

7. **Publicness and Public Engagement in Academic Leadership**

- In which public spheres do you perceive that issues important to you are being addressed? In what ways are you able to influence these spheres, and how can you contribute to making these issues more visible and impactful?
- How would you describe your private and professional opportunities to act on behalf of the issues you consider important in society? In your view, how is your social media non-use reflected in your capacity to engage with these issues?
- How do you define the publicness (julkisuus in Finnish) in contemporary society?
- Which communities that are important to you do you feel you belong to?
- In what ways are you able to influence issues that are meaningful in your life?
- Where do the topics of your everyday conversations typically originate?

8. **Looking Toward the Future**

- How do you see your relationship with social media evolving in the future? What are your views on your opportunities for participation and action?
- Is there anything else you would like to highlight regarding the themes discussed in this interview?
- Thank you for taking part in this interview.
